# Hermaphrodites and parasitism: size-specific female reproduction drives infection by an ephemeral parasitic castrator

**DOI:** 10.1038/s41598-019-55167-x

**Published:** 2019-12-13

**Authors:** Caitlin R. Fong, Armand M. Kuris, Ryan F. Hechinger

**Affiliations:** 1 University of California, Santa Barbara—Department of Ecology, Evolution, and Marine Biology, and Marine Science Institute, Santa Barbara, California, 93106 USA; 2 Scripps Institution of Oceanography—Ocean Biosciences Program, University of California, San Diego, La Jolla, California, 92093 USA

**Keywords:** Ecological epidemiology, Population dynamics

## Abstract

Sex can influence patterns of parasitism because males and females can differ in encounter with, and susceptibility to, parasites. We investigate an isopod parasite (*Hemioniscus balani*) that consumes ovarian fluid, blocking female function of its barnacle host, a simultaneous hermaphrodite. As a hermaphrodite, sex is fluid, and individuals may allocate energy differentially to male versus female reproduction. We predicted the relationship between barnacle size and female reproductive function influences the distribution of parasites within barnacle populations. We surveyed 12 populations spanning ~400 km of coastline of southern California and found intermediate-sized barnacles where most likely to be actively functioning as females. While it is unclear why larger individuals are less likely to be actively reproducing as females, we suggest this reduced likelihood is driven by increased investment in male reproductive effort at larger sizes. The female function-size relationship was mirrored by the relationship between size and parasitism. We suggest parasitism by *Hemioniscus balani* imposes a cost to female function, reinforcing the lack of investment in female function by the largest individuals. Within the subset of suitable (=female) hosts, infection probability increased with size. Hence, the distribution of female function, combined with selection for larger hosts, primarily dictated patterns of infection.

## Introduction

Sex can drive patterns of parasitism in host populations for two overarching reasons. First, behavioral differences between males and females can lead to differences in encountering parasite transmission stages [e.g^1–3^.]. Second, males and females can differ regarding their compatibility as hosts after encounter^4^. An interesting twist occurs when sex is not fixed. In hermaphroditic species, individuals may allocate variable amounts of energy to male or female function^5^. In such cases, individuals may experience sex-based differences in levels of parasitism in a single lifetime.

*Hemioniscus balani* is a parasitic isopod that specialises on barnacle ovarian fluid and blocks female reproduction^6–8^. This protandrous isopod infects at least 14 species of hermaphroditic barnacles and has a cosmopolitan distribution^6–9^. Infection occurs when a highly mobile cryptoniscus larva finds an appropriate barnacle. This cryptoniscus is a large (>1 mm) and highly mobile isopod. The cryptoniscus enters the barnacle and attaches to and feeds on the ovaries, draining ovarian fluid. This results in suppression of female function while male function is retained^6,9,10^. The parasite metamorphoses from male to female inside the barnacle host, where it is fertilized by another cryptoniscus stage which then leaves that host^6,9,10^. Multiple infections are infrequent^11^. The parasite matures, releases its offspring, and dies, after which the host recovers female reproductive function^6^; mortality to the host has never been documented. The parasite offspring enter the plankton, likely attaching to a copepod intermediate host, before infecting the barnacle host to mature and complete their life cycle^6,9,10^. Mathematical models suggest that infection (time from entry to life cycle completion) lasts ~10 days^12^; thus, the parasite is very short lived compared to the barnacle host. This isopod is an ephemeral, semelparous parasitic castrator—a distinctive strategy with no other examples known to us (see^13,14^ for reviews of parasitic castration).

*Chthamalus fissus* is a species of barnacle frequently infected by *Hemioniscus balani*. Like many barnacles, *C. fissus* is a protandrous hermaphrodite—individuals begin their lives as males and, with increasing age and size, allocate more energy to female function to become simultaneous hermaphrodites^5,15–18^. Thus, the amount of energy barnacles allocate to female function can vary with age. The parasite’s specialization on female ovarian fluid, combined with the hosts’ variable allocation to female function, makes this a useful and interesting system to study the intersection between parasitism and female sex allocation.

In this study, we use a survey approach to assess the relationship between barnacle size, female reproduction, and parasitism. We hypothesize that patterns of infection by *H. balani* mirror variation in barnacle female function because the parasite is an ovary specialist. However, among suitable (=female) hosts, we hypothesize infection risk increases with body size. This could happen for two reasons, though we are not able to discriminate between the two. First, larger barnacles are larger targets, and should be more likely to encounter infectious stages. Second, parasites might prefer, and actively infect, larger hosts with more resources (e.g.^13,19–21^). To test these hypotheses, we surveyed barnacles for size, female reproductive function, and parasitism by *H. balani* from 12 populations at 6 localities along the Southern California Bight.

## Methods

*Chthamalus fissus* is reproductive year-round, with peak brooding in spring through summer^21^. Female reproductive functionality can fluctuate as a product of the brooding cycle and food availability, while male functionality is always present once maturity is reached^22^. We use the presence of female functionality as a binary approximation of female reproductive effort because these barnacles can complete an entire brood cycle in ~2 weeks, and ovary development and brooding can overlap [22, *pers obs*]. However, these barnacles have regressed ovaries when not reproducing as female, or when between broods^22^, making the absence of ovary development or brooding eggs an indicator that the individual is not currently investing in female reproductive effort. Thus, we infer that the fraction of the population investing in female reproduction at any given size provides an index of the female reproductive effort of that size class.

**Figure 1:**
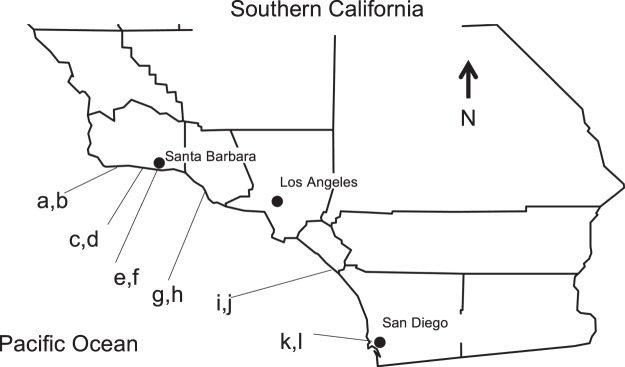
Locations of the 12 survey sites, which were at six localities spread throughout the Southern California Bight. Localities included Gaviota (**a**,**b**), Goleta (**c**,**d**), Santa Barbara (**e**,**f**), Ventura (**g**,**h**), San Clemente (**i**,**j**), and La Jolla (**k**,**l**), the site lettering corresponds to the site panels in Figs. 2 and S1.

Over three days (16–18 September 2013), we collected barnacles from 2 habitat types, natural rock and pier pilings, at 6 localities spread throughout the Southern California Bight for a total of 12 populations (Fig. 1). At each locality, we collected barnacles from the two habitats to assess possible differences between these habitats because previous research indicates naturally occurring versus artificially constructed habitats ecologically differ (e.g., in species numbers, abundances, and diversity), possibly due to differences in water flow^23,24^. Localities were chosen for accessibility and availability of both habitat types. To minimize tidal differences and differences in encounter rate, barnacles were collected in a stratified random design from the lower 10 cm of their elevational range. We collected all barnacles encountered in 10 haphazardly placed circular 11.34 cm^2^ cores. Barnacles likely assess mating group size through physical contact with their penises; thus, our quadrat size should encompass at least one mating group, and likely parts of more along the edges (scaling based on^25^). Barnacles were frozen immediately until dissection.

In the laboratory, all barnacles were thawed, identified, and dissected. We ensured each dissected barnacle belonged to the genus *Chthamalus* based on plate arrangement. *C. fissus* is the most common *Chthamalus* species in southern California^26,27^. We cannot exclude the possibility that another *Chthamalus* species, *C. dalli*, was present in our samples. However, such an event would likely be unimportant, given its rarity. Further, the two species are similar in size, biology, and ecology^26^ and *Hemioniscus balani* infects both species^9^. Hence, we refer to the host as *C. fissus*.

For each barnacle, length was measured as the widest shell base diameter to the nearest 0.25 mm. Barnacles were recorded as infected or uninfected, and as non-reproductive or reproductive, based on barnacle female reproductive function, where reproductive individuals had ripe ovaries (as indicated by yellow/orange fluid within the ovary), developing eggs, or oviposited eggs. The cryptoniscus is >1 mm in length, simplifying detection of early infections. We only included barnacles ≥1 mm, avoiding barnacles that are typically pre-reproductive (^16^; CRF *personal observations*).

We determined whether the size frequency distribution of infected hosts was a non-random subset of the size frequency distribution characterizing the entire barnacle population using a Kolmogorov-Smirnoff test (infected versus whole sample).

We examined the influence of size, habitat type, and locality on the probability of female function and infection using logistic regression. We implemented logistic regression as generalized linear mixed models with a binomial error distribution and a logit link^28^. To permit hump-shaped relationships with size, we included size along with a quadratic term (using centered-size data to preclude collinearity problems)^29^. Because we sampled each of the two habitat types at each locality (factors were crossed), and we were interested in whether there were any consistent differences among habitats or localities, we included these factors as fixed effects along with their interactions with each other and with size. We modelled individual replicate core as a random effect to account for the potential lack of independence of barnacles within individual cores. For this “CoreID” random effect, we included both random intercepts and random slopes (with size), which permits the most accurate parameter estimates for the fixed effects^30^.

We first fit global models including all terms of interest. We used AIC values to determine whether simpler models would be favored, with a particular emphasis on whether maintaining the quadratic-size term was favored, indicating unimodality. We focused on the top model, but examined all favored models (i.e., those within 2 AIC values of the top model^31^) to ensure general consistency in results.

We examined the probability of female function for two sets of the data. We first did so only among uninfected barnacles. However, disproportionate infection of barnacles with female function would lead to underestimating this probability. For instance, in the extreme case of 100% infection of barnacles with female function, there would be zero probability of female function. Hence, we also examined the probability of female function by including infected barnacles as “reproductive females”. This is sensible given the parasite’s specialization on female ovaries, which necessarily implies that the barnacle was a functional female at time of infection. The predicted values from both analyses were very similar (*r* = 0.89, *p* < 2.2 × 10^−16^, *n* = 6,018) as expected given the typically low prevalence of infection. We therefore present the results counting infected individuals as barnacles with female function, because that likely provided the best representation of the pattern.

We examined the probability of infection among all barnacle individuals, and, separately, for the subset of barnacles in the “susceptible class,” defined as only uninfected, reproductive barnacles, and infected barnacles (i.e., excluding uninfected barnacles that had zero female function).

We used the regression equations from the favored models to extract the host size corresponding to the maximum probabilities of female function and infection for each site. We compared the size of maximum probability of female function to the maximum probability of infection at each site using a paired t-test.

We ran all analyses in R v. 3.5.2^32^ and JMP Pro v. 12 (SAS Institute, Inc.). We used the glmer function in the Lme4 package v. 1.1-21^33^ for the logistic regressions. We assessed model adequacy using Pearson goodness of fit tests (suitable, given the high replication at each size), plots of Pearson residuals versus predicted values for the global models^28,31,34^, and, for the favored models, by comparing model predicted values to observed data.

## Results

**Figure 2:**
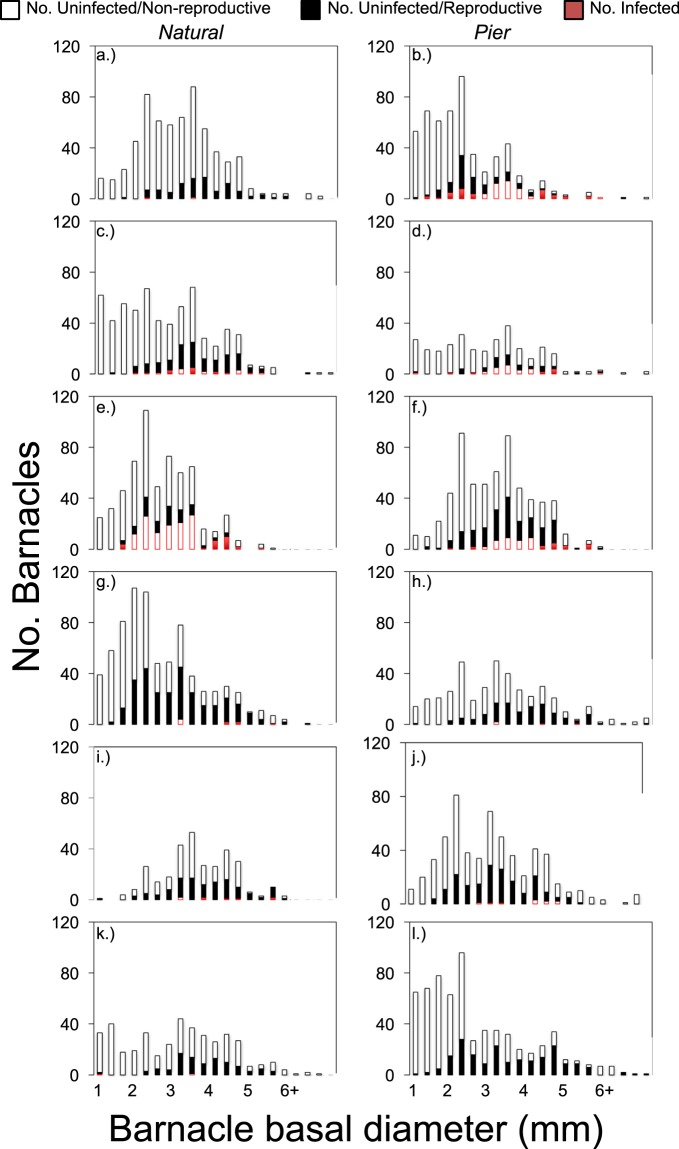
Size-frequency distributions of the barnacle populations at the 12 study sites, which were located throughout the Southern California Bight. Bars indicate number of barnacles that were uninfected/non-reproductive (white), reproductive (black), and infected (red). Natural rock habitats are on the left, while pier habitats are on the right. Sites are ordered from north to south: Gaviota (**a**,**b**), Goleta (**c**,**d**), Santa Barbara (**e**,**f**), Ventura (**g**,**h**), San Clemente (**i**,**j**), and La Jolla (**k**,**l**).

We dissected 6,381 barnacles, of which 362 were infected, providing a regional prevalence (percentage infected) of 5.7% [5.1–6.3 95% CI] (Table 1). However, prevalence varied substantially between sites and ranged from 0.0 to 23.9%. The size frequency distribution of infected barnacles was significantly different from uninfected barnacles at all sites at which there were at least 10 infections (Table 1). Thus, the distribution of infected individuals was not a random subset of the host population (Fig. 2).

**Table 1:** Sites sampled over 3 days in 2013, number of *Chthamalus fissus* barnacles examined (N), number infected by of *Hemioniscus balani* and parasitism prevalence.

SITE	KS P-VALUE	# INFECTED	N	PREVALENCE (%) [95% CI]*
Gaviota Rock	0.9973	2	629	0.3 [0.1–1.2]
Gaviota Pier	**<0.0001**	75	536	14.0 [11.3–17.2]
Goleta Rock	**0.0005**	25	615	4.1 [2.8–5.9]
Goleta Pier	**0.0003**	31	201	15.4 [7.3–14.2]
Santa Barbara Rock	**<0.0001**	143	598	23.9 [20.1–27.5]
Santa Barbara Pier	**<0.0001**	52	614	8.5 [6.5–10.9]
Ventura Rock	**0.0003**	10	743	1.3 [0.7–2.5]
Ventura Pier	0.4317	4	410	1.0 [0.4–2.5]
San Clemente Rock	0.4846	8	308	2.6 [1.3–5.0]
San Clemente Pier	**0.0198**	10	572	1.7 [1.0–3.2]
Scripps Rock	0.8701	2	413	0.5 [0.1–1.7]
Scripps Pier	—	0	642	0.0 [0–0.6]

Kolmogorov-Smirnoff (KS) test probabilities comparing the size frequency distribution of infected and uninfected barnacles at each site. N also indicates barnacle density (number in 10 randomly placed circular 11.34 cm^2^ cores).

*Mean values of prevalence are reported followed by 95% confidence interval, calculated using the score method^49^.

**Figure 3:**
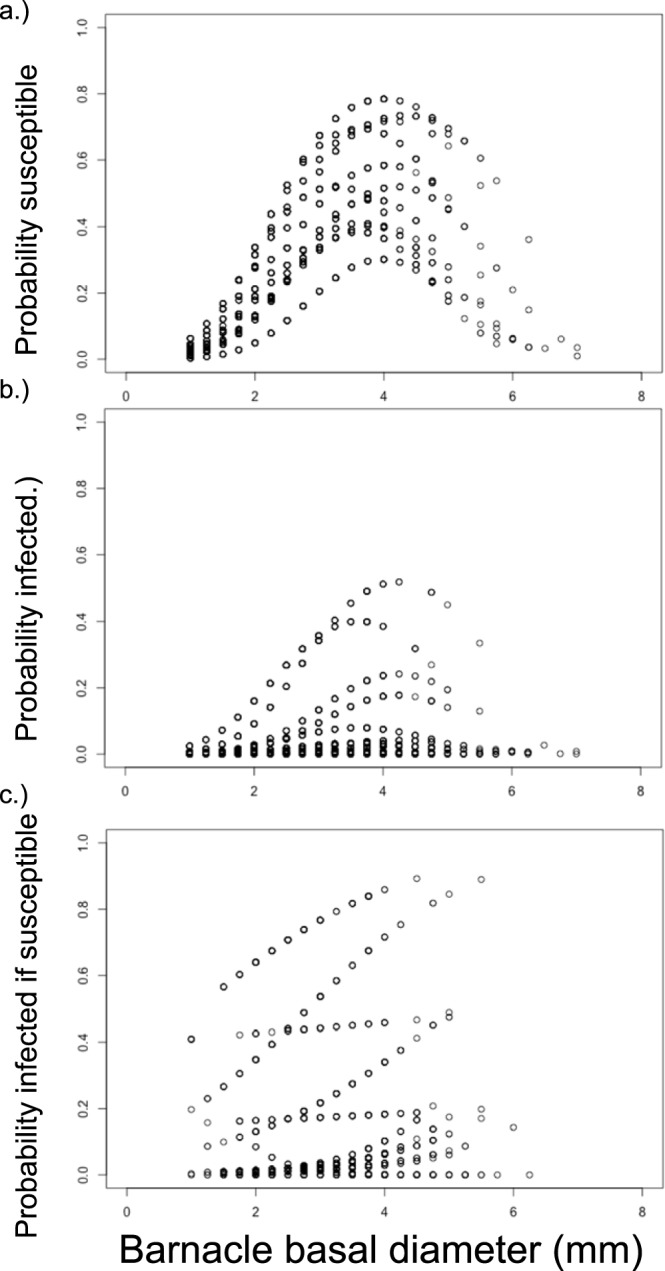
Probabilities from the favored models relating size to (**a**) probability of being susceptible (reproductive + infected), (**b**) probability of being infected, and (**c**) the probability of being infected given susceptibility (reproductive + infected).

All favored models for female function included barnacle host size, habitat type, and locality, and several interactions between these variables (Table 2). These interactions reflected variation among localities and habitats in the specific shape of the relationship between reproduction and size. Despite this population-level variation, the probability of female reproduction was consistently unimodal, with intermediate-sized barnacles always having the highest probability of being female (Fig. 3). While localities did vary in overall levels of parasitism, their effects also varied by habitat type. Further, one habitat type did not consistently have a higher probability of female function than did the other (indicated by the weak main effect of habitat type, but the retention of the locality*habitat type in all favored models).

**Table 2:** Summary of the generalized linear mixed models and AIC used for model selection.

model	locality	habitat	size	locality* size	habitat* size	habitat* locality	habitat* locality* size	size*size	size* size* locality	size* size* habitat	size* size* habitat* locality	AIC
**(A) Model selection for the probability of being susceptible to infection**												
**1**	**x**	**x**	**x**	**x**	**x**	**x**	**x**	**x**		**x**		**6519.9**
2	x	x	x	x	x	x	x	x				6520.7
3	x	x	x	x	x	x	x	x	x	x		6521.6
4	x	x	x	x	x	x	x	x	x	x	x	6521.7
5	x	x	x			x		x				6523.3
6	x	x	x		x	x		x				6524.1
7	x	x	x	x		x		x				6528.5
8	x	x	x	x	x	x		x				6528.7
9	x	x	x			x						6724.8
10	x	x	x	x	x	x	x					6726.5
11	x	x	x	x		x						6731.3
12	x	x	x	x	x	x						6733.3
13	x	x	x	x								6761.5
**(B) Model selection for the probability of being infected**												
**1**	**x**	**x**	**x**		**x**	**x**		**x**				**2010.6**
2	x	x	x	x	x	x		x				2010.7
3	x	x	x	x		x		x				2012.6
4	x	x	x	x	x	x	x	x				2013.6
5	x	x	x			x		x				2014.8
6	x	x	x	x	x	x	x	x		x		2015.3
7	x	x	x	x	x	x	x	x	x	x		2023.7
8	x	x	x	x	x	x	x	x	x	x	x	2031
9	x	x	x			x						2077.1
10	x	x	x	x		x						2079
11	x	x	x	x	x	x						2080.9
12	x	x	x	x	x	x	x					2084.3
13	x	x	x	x								2152.9
**(C) Model selection for the probability of being infected given susceptibility**												
**1**	**x**	**x**	**x**	**x**		**x**						**1217.1**
2	x	x	x	x	x	x						1217.1
3	x	x	x	x	x	x		x				1221
4	x	x	x	x		x		x				1221.2
5	x	x	x	x	x	x	x					1221.7
6	x	x	x			x						1221.7
7	x	x	x	x	x	x	x	x				1225.5
8	x	x	x		x	x		x				1226.7
9	x	x	x	x	x	x	x	x		x		1227.4
10	x	x	x			x		x				1229
11	x	x	x	x	x	x	x	x	x	x		1235.8
12	x	x	x	x	x	x	x	x	x	x	x	1243.9
13	x	x	x	x								1269.9

The two favored models for infection probability also included barnacle host size, habitat type, and locality, and some two-way interactions (Table 2). Here too, infection probability was consistently a unimodal relationship with host size (the quadratic term was not only maintained in the two favored models, but in the top 8 models). That is, intermediate-sized barnacles had the highest probability of infection, irrespective of population (Fig. 3). The specific shape of the unimodal relationship between infection probability and size was more consistent than that of female function among populations, with only the habitat*size interaction being favored. However, localities and habitats did vary in overall infection risk (e.g., the height of the curves)—with one population even completely lacking infection, and the strength of their effects depended on one another (the habitat*locality interaction maintained in the top models).

The favored model for infection probability within the class of susceptible barnacles included site, habitat, and size (Table 2). The quadratic size term was not retained in the top models, reflecting the general increase of the probability of infection with size (Fig. 2). This general increase with size occurred at most populations, though the height and shape of the curves varied among localities (locality and locality*size terms being retained in each favored model). Here too, habitat had an inconsistent effect among localities (locality*habitat term retained in top two models).

## Discussion

**Figure 4:**
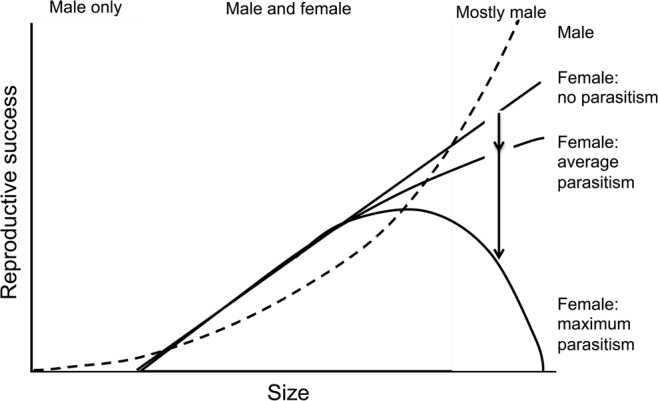
Conceptual diagram of reproductive success as relates to barnacle size. Along the top we have indicate our hypothesized sex allocation. Reproductive success for females is depicted as a solid black line under 3 scenarios: no parasitism, average parasitism, and maximum parasitism. A dashed black line represents male reproductive success.

Intermediate sized barnacles were most likely to have female reproductive function and most likely to be infected. We consistently detected that parasitism tracked the distribution of female function, consistent with the known biology of *H. balani*, which specialises on female function of the host and has an active searching stage that should permit it to find preferred barnacle hosts. For *Chthamalus fissus*, male function reaches maturity before female function^22^; thus, small barnacles are only male. Once female reproductive maturity is attained, barnacles have both female and male function^35,36^. Theoretical models predict at maturity, allocation is predominantly to female function, with allocation to male function increasing with sperm competition to a maximum of 50%^5^. There is a linear relationship between barnacle size and number of eggs (a proxy for reproductive success) for *C. fissus*^22^. This linear relationship between size/age and female function is generally assumed in simultaneous hermaphrodites^37^. Thus, both models and empirical data support an increased likelihood of female reproductive function and female reproductive success with increased sized (Fig. 4).

It is unclear why larger individuals are less likely to be actively reproducing as females. We suggest this reduced likelihood is driven by increased investment in male reproductive effort at larger sizes (Fig. 4). We find it implausible that the largest individuals do not invest at all in reproduction, as the life history of most species does not involve a substantial post-reproductive growing period^38,39^. We also find it unlikely that physical damage to female reproductive organs due to sequential infections made investment in female function impossible, because this unimodal relationship was generalizable across populations with low and high parasitism. We suggest male reproductive success may increase with barnacle length if sperm production and penis length increases with length. Research indicates sperm production can be plastic; an androdiecious barnacle shows reduced allocation to sperm production in solitary versus gregarious populations^40^. Our species is a gregarious settler with large mating groups and plasticity in sperm production may result in increased allocation in large groups. In this system, male reproductive success is limited by penis length. Thus, large individuals may have increased reproductive success by functioning primarily as male, as in other hermaphroditic mating systems^41–43^.

We posit parasitism by *Hemioniscus balani* imposes a cost to female function, reinforcing the lack of investment in female function by the largest individuals. There are some parallels to this shift in sex allocation in another system. A protogynous sequentially hermaphroditic reef fish can be infected by a myxozoan parasite that renders the eggs infertile. In this system, infected individuals transition to male at smaller sizes, suggesting plasticity in response to parasitism^44^. Thus, parasites can impose substantial fitness costs, altering patterns of female reproduction.

Within the subset of appropriate (=female) hosts, parasitism increased with size. We implicate active parasite choice as a driver of this pattern. The actively searching stage of a parasite can be highly selective; for example, adult female wasps select hosts for their parasitoid offspring based on a range of host characteristics including size^45,46^. Larger hosts typically result in increased body size/reproductive output for parasitic castrators^13^. This is the case for *H. balani*, as parasite body size and fecundity increase in larger hosts^11^. The highly mobile cryptoniscus searching stage of *H. balani* presumably has the physical and behavioral capacity to select a host. While parasite prevalence often increases with host size because cumulative infection risk is higher for older/larger individuals^3^, this is unlikely here because the parasite is semelparous and short-lived. We cannot exclude increased encounter rate as a driver—larger hosts are larger targets and thereby have increased encounter rates, and rejection of a host might pose to great of a cost to enable choice. However, while experimental evidence would be more compelling, we find active host choice the most likely mechanism for the increased parasitism of larger, susceptible hosts, due to the searching capability of *H. balani* and the fitness gains from infecting a larger host.

There is another useful system for comparison that may further our understanding size, parasitism, and sex allocation. Other species of barnacles are infected by rhizocephalan parasites in the family Chthamalophilidae. This family of parasites infects barnacles and takes up space in the brood cavity^47,48^. While sexual function is retained, the brood cavity does become filled, which may limit brood size^47,48^. This cost to female reproduction may influence mating groups and sex allocation. A key difference between *H. balani* and Chthamalophilidae is *H. balani* is semelparous while Chthamalophilidae are a more typical parasite with a longer life span. Thus, comparisons of the effects of sex allocation may be particularly useful for elucidating underlying principles of sex allocation, size, and parasitism.

## Supplementary information

Supplement

## Data Availability

The datasets generated for the current study are available from the corresponding author on reasonable request.
