# Supplementary material for: Hermaphrodites and parasitism: size-specific female reproduction drives infection by an ephemeral parasitic castrator
Source: Sci Rep. 2019 Dec 13;9:19121. doi: 10.1038/s41598-019-55167-x (PMC6911060; doi:10.1038/s41598-019-55167-x)
Supplement: Supplementary file 1 — Supplement [file 41598_2019_55167_MOESM1_ESM.docx]

SUPPLEMENT

Hermaphrodites and parasitism: size-specific female reproduction drives infection by an ephemeral parasitic castrator

Caitlin R. Fong

Armand M. Kuris

Ryan F. Hechinger

Table S1 Summary of the generalized linear mixed models and AIC used for model selection
